# Comparison of scoring methods for the detection of causal genes with or without rare variants

**DOI:** 10.1186/1753-6561-5-S9-S49

**Published:** 2011-11-29

**Authors:** Markus Scholz, Holger Kirsten

**Affiliations:** 1 Institute for Medical Informatics, Statistics, and Epidemiology (IMISE), Universität Leipzig, Härtelstrasse 16-18, 04107 Leipzig, Germany; 2 LIFE Center (Leipzig Interdisciplinary Research Cluster of Genetic Factors, Phenotypes, and Environment), Universität Leipzig, Leipzig, Philipp-Rosenthal-Strasse 27, 04103 Leipzig, Germany; 3 Translational Center for Regenerative Medicine, Universität Leipzig, Philipp-Rosenthal-Strasse 55, 04103 Leipzig, Germany; 4 Department for Cell Therapy, Fraunhofer Institute for Cell Therapy and Immunology, Perlickstrasse 1, 04103 Leipzig, Germany

## Abstract

Rare causal variants are believed to significantly contribute to the genetic basis of common diseases or quantitative traits. Appropriate statistical methods are required to discover the highest possible number of disease-relevant variants in a genome-wide screening study. The publicly available Genetic Analysis Workshop 17 data set consists of 697 individuals and 24,487 genetic variants. It includes a simulated complex disease model with intermediate quantitative phenotypes. We compare four gene-wise scoring methods with respect to ranking of causal genes under variable allele frequency thresholds for collapsing of rare variants and considering whether or not rare variants were included. We also compare causal genes for which the ranks differ clearly between scoring methods regarding such characteristics as number and strength of causal variants. We corroborated our findings with additional simulations. We found that the maximum statistics method was superior in assigning high ranks to genes with a single strong causal variant. Hotelling’s *T*^2^ test was superior for genes with several independent causal variants. This was consistent for all phenotypes and was confirmed by single-gene analyses and additional simulations. The multivariate analysis performed similarly to Hotelling’s *T*^2^ test. The least absolute shrinkage and selection operator (LASSO) analysis was widely comparable with the maximum statistics method. We conclude that the maximum statistics method is a superior alternative to Hotelling’s *T*^2^ test if one expects only one independent causal variant per gene with a dominating effect. Such a variant could also be a supermarker derived by collapsing rare variants. Because the true nature of the genetic effect is unknown for real data, both methods need to be taken into consideration.

## Background

For many common diseases and quantitative traits investigators have observed a gap between heritability estimates and the variance explained by common genetic variants discovered in genome-wide association studies. Rare and low-frequency causal genetic variants have been suggested to fill a significant part of this gap [1]. High-throughput sequencing for the identification of rare variants is technically feasible now. However, given the size of most current studies, the low frequency of rare variants heavily affects the power of single-marker association tests. Hence it is necessary to either pool rare variants or combine them with common variants [1]. The Genetic Analysis Workshop 17 (GAW17) data set allows comparison of genome-wide screening methods for both common and rare variants. These methods should be optimized regarding the enrichment of true positive variants in gene lists selected for future replication studies.

In this paper, we compare four scoring methods on the basis of the GAW17 data set of unrelated individuals for different analysis parameters (allele frequency cutoffs for the definition of rare variants and use of rare variants): the maximum statistics method, the LASSO (least absolute shrinkage and selection operator) method, Hotelling’s *T*^2^ test, and multivariate analysis. In contrast to single-marker testing, we focus on identification of causal genes using gene-wise scores.

We investigate under what conditions each of the methods performs better. For this purpose, we rank all genes on the basis of their scores, and we are interested in the high ranking of genes that contain causal variants. More precisely, we pursue answers to the following questions: (1) Are there differences in the performance of scoring methods, and if so, why? (2) Does the cutoff for allele frequency of rare variants have an influence on the performance? (3) Does inclusion of rare variants improve the search for causal variants in our data set? These analyses were accompanied by additional simulations.

## Methods

### Data set

Our study is based on the GAW17 data set, as described elsewhere [2]. We were aware of the simulation model and analyzed all 200 simulation data sets of unrelated individuals.

### Data pre-analysis, test statistics, and models

We considered two alternative allele frequency cutoffs (1% and 5%) to define a variant as rare. We created four different data sets for analysis: CR1% and CR5% include all common variants with frequencies above 1% and 5%, respectively, and incorporate gene-wise collapsing of nonsynonymous rare variants into a single supermarker, as described elsewhere [3]. CR1% consists of 7,529 variants with 2,451 genes, and CR5% consists of 4,617 variants with 2,124 genes. For the definition of *gene-wise*, we applied the gene annotations provided by GAW17 [2]. Data sets C1% and C5% are identical to CR1% and CR5%, respectively, but exclude all rare variant supermarkers. C1% consists of 6,356 variants with 2,208 genes, and C5% consists of 3,132 variants with 1,473 genes. Markers with fewer than five minor alleles were eliminated to stabilize regression modeling. Linkage disequilibrium of rare variant supermarkers with common variants was low (,  for causal markers of CR1%, and ,  for causal markers of CR5%).

Using linear and logistic regression techniques, we analyzed the following three endpoints: traits Q1, Q2, and affected status (AFF) adjusted for Q1, Q2, and Q4. Analysis of Q1 was adjusted on Q2 and vice versa to reduce variance as much as possible according to the simulated model. In addition, all three endpoints were adjusted for age, sex, and smoking status. We assumed an additive genetic model throughout.

The maximum statistics method was performed by calculating all marginal models of a gene, including those of the rare variant supermarker, if applicable. The maximum of the *t*-statistic was used as the score for a gene. For Hotelling’s *T*^2^ test, marginal models were combined using Hotelling’s *T*^2^ calculated on the basis of the correlation matrix of the markers. Multivariate analysis was calculated for each gene by performing a likelihood ratio test between the model with all genetic markers of a gene and the null model without any genetic effects. For the LASSO method, we applied the R routine glmnet with standard attributes of version 1.5.2 (R 2.11.0, http://www.r-project.org). The tuning parameter was determined by means of cross-validation, as recommended by Friedman et al. [4]. The shrunken model was compared with the null model using the likelihood ratio test. Only genetic markers were shrunk. Genes were ranked according to their summary statistics. Analyses were repeated for each of the 200 simulation replicates in the GAW17 data set.

### Assessment of regression analysis results and additional simulations

We defined cutoff values of 10, 20, 50, 100, 200, and 500 best-ranked genes for inclusion in a potential replication study. The average number of phenotype-specific causal genes in each of these gene lists was calculated over all 200 simulations.

To determine which of the analysis parameters (method, cutoff for rare variants, inclusion of rare variants) were favorable, we compared the numbers of identified causal genes between scenarios using multivariate mixed model analysis (SAS 9.1.3). Method, cutoff for rare variants, use of rare variants, and the cutoff-use interaction were treated as fixed factors. The simulation scenario was treated as a random factor. This analysis was done separately for all gene list cutoffs and phenotypes.

To identify conditions that influence performance of the scoring methods, we analyzed the following parameters for each causal gene: number and percentage of causal and noncausal common and rare variant supermarkers; effect sizes of the supermarkers (*β* coefficient multiplied by the standard deviation of the allele frequency); frequencies and effect sizes of the second strongest variant; and relative size of the second strongest effect. We compared these characteristics between genes for which the methods performed differently as defined by a ratio of median ranks greater than 1.1 or smaller than 0.9. A gene was included in this analysis only if its rank was greater than 1,000 for at least one method. Otherwise, it was considered undetectable.

In addition, we simulated a set of 2,000 genes without genetic effect containing two, three, five, or seven common or collapsed markers, each with allele frequencies drawn from the uniform distribution. For an additional gene we assumed a causal effect of one, two, or three (where applicable) markers with a specific allele frequency (1%, 5%, 10%, 20%, or 40%) and odds ratio (1.2, 1.3, 1.5, or 1.7). This causal gene was simulated 1,000 times. We considered samples of *N* = 300 or *N* = 1,000 case subjects and an equal number of control subjects. Ranks of the causal gene were compared between methods.

## Results

### Analysis of best-ranked genes for analysis scenarios

The observed frequencies of causal genes were significantly higher than expected at random for all analysis scenarios except for the C5% data set at the gene list cutoff of *N* = 500. Using, for example, a cutoff of 200 top genes and averaging over all 200 replicates, we discovered 2.2–3.6, 2.6–6.1, and 2.3–3.2 causative genes for Q1, Q2, and AFF, respectively, depending on the analysis setting. Scoring method, cutoff for rare variants, and inclusion of rare variant supermarkers significantly influenced the performance of analyses.

The results of multivariate analyses are summarized in Table 1. A 5% allele frequency cutoff was advantageous for Q1, for selection of a low number of genes, and for AFF throughout but not for Q2. Analysis of rare variant supermarkers strongly improved the power of Q1 and Q2 analyses but not the power of AFF analysis. For Q1 and Q2 but not for AFF there was a strong interaction of allele frequency and use of rare variants, favoring the 1% cutoff if rare variants were excluded. As expected, Hotelling’s *T*^2^ test and multivariate analysis performed similarly. The maximum statistics and LASSO methods also mostly showed similar performance. Either the maximum statistics method or Hotelling’s *T*^2^ test outperformed the other methods in most scenarios. Compared to Hotelling’s *T*^2^ test, the maximum statistics method performed better for longer gene lists. This effect was more pronounced for Q2 and AFF than for Q1.

**Table 1:** Results of mixed model analysis of the ranked gene lists for all phenotypes

Phenotype	Gene list cutoff (*N*)	MS vs. HT	MS vs. MV	MS vs. LA	HT vs. MV	HT vs. LA	MV vs. LA	AF = 1% vs. AF = 5%	CV vs. RV	IA AF = 1% and CV
Q1	10	−0.27***	−0.25***	−0.15***	0.02	0.13***	0.10***	−0.18***	−0.73***	0.43***
	20	−0.19***	−0.18***	−0.11***	0.01	0.08***	0.07***	−0.10***	−0.71***	0.45***
	50	−0.14***	−0.12***	*−*0.03	0.02	0.11***	0.09***	0.01	−0.66***	0.51***
	100	*−*0.05	*−*0.03	0.06*	0.02	0.11***	0.09**	0.10***	−0.77***	0.76***
	200	*−*0.01	0.01	0.11***	0.02	0.12***	0.10***	0.21***	−1.09***	1.07***
	500	0.08*	0.09**	0.13***	0.01	0.05	0.04	0.42***	−2.00***	1.85***
Q2	10	*−*0.04	*−*0.06	0.03	*−*0.02	0.07*	0.09**	0.14***	−0.67***	0.32***
	20	*−*0.03	*−*0.03	*−*0.04	*−*0.00	*−*0.01	*−*0.01	0.14***	−1.05***	0.46***
	50	0.08	0.07	*−*0.01	*−*0.01	−0.09*	−0.08*	0.26***	−1.69***	0.56***
	100	0.14**	0.13**	*−*0.00	*−*0.01	−0.14**	−0.14**	0.22***	−2.32***	0.72***
	200	0.11*	0.12*	0.04	0.01	*−*0.07	*−*0.08	0.03	−3.14***	1.13***
	500	0.25***	0.25***	0.11*	0.00	−0.13*	−0.13*	0.18***	-3.88***	1.19***
AFF	10	0.03	*−*0.01	0.04**	−0.04*	0.02	0.06***	−0.15***	0.02	*−*0.03
	20	0.09***	0.08***	0.05**	*−*0.01	−0.04*	*−*0.03	−0.16***	0.03	*−*0.03
	50	0.16***	0.14***	0.03	*−*0.02	−0.13***	−0.11***	−0.14***	0.03	*−*0.05
	100	0.34***	0.29***	0.11***	*−*0.05	−0.23***	−0.18***	−0.13***	0.14***	−0.16***
	200	0.44***	0.34***	0.07	−0.10*	−0.37***	−0.27***	−0.20***	0.21***	−0.25***
	500	0.69***	0.60***	0.15***	−0.09*	−0.54***	−0.45***	−0.40***	0.39***	−0.33***

*N* is the cutoff value of the ranked gene lists for inclusion in a future replication study. MS, maximum statistics method. HT, Hotelling’s *T*^2^ test. MV, multivariate analysis. LA, LASSO method. AF, cutoff for allele frequency denoting rare variants. CV, analysis of common variants alone. RV, analysis of both common and collapsed rare variants. IA = interaction term. For the comparisons, values denote the average difference in number of detected causal genes. Negative numbers indicate a lower number of causal genes under the first setting. Significant positive and negative effects are in boldface and italics, respectively.

* *p* < 0.05.

** *p* < 0.01.

*** *p* < 0.001.

### Detailed characterization of detectable causal genes and additional simulations

To understand this behavior in more detail, we determined the median ranks of all causal genes for each data set and each method over all 200 simulation replicates.

Genes that were better detected using the maximum statistics method compared to Hotelling’s *T*^2^ test were characterized by a higher number of markers per gene, a lower relative frequency of causal variants, and often a lower effect size of the strongest variant. Genes that were better detected using the maximum statistics method compared to the LASSO method also showed a lower effect size of the strongest variant and weaker relative effects of the second strongest variant. An overview of all characteristics can be found in Table 2.

**Table 2:** Characteristics of genes for which gene-wise scoring methods performed differently

	MS vs. HT						MS vs. LA					
	
	CR1%			CR5%			CR1%			CR5%		
	
	MS better	HT better	*p*-value	MS better	HT better	*p*-value	MS better	LA better	*p*-value	MS better	LA better	*p*-value
Rank (MS)/rank (other method)	0.53 (0.14)	2.4 (2.56)		0.56 (0.15)	1.43 (0.31)		0.7 (0.23)	3.09 (3.81)		0.72 (0.58)	1.41 (0.34)	
Genes with better ranks	13/27	8/27		12/29	13/29		7/27	4/27		7/29	5/29	
Markers per gene	**5.8 (2.6)**	**2 (0.8)**	<0.001	**4.8 (2.1)**	**1.5 (0.9)**	<0.001	4.3 (2.7)	5.8 (4.9)		4.2 (2.2)	2.2 (1.1)	
Causal SNPs per gene (number)	1.3 (0.5)	1.4 (0.7)		1.1 (0.3)	1.2 (0.4)		1.4 (0.5)	2.5 (1.3)		1.2 (0.4)	1.6 (0.5)	
Causal SNPs/gene (%)	**25.6 (11.6)**	**72.9 (29.5)**	0.002	**26 (10.7)**	**88.5 (21.9)**	<0.001	42.8 (27.9)	68.4 (40)		39.6 (32)	80 (27.4)	
Frequency-adjusted effect size of strongest variant	0.038 (0.024)	0.044 (0.023)		0.025 (0.02)	0.048 (0.025)	0.02	0.033 (0.022)	0.05 (0.023)		0.025 (0.022)	0.066 (0.028)	0.03
Frequency of strongest variant	0.047 (0.077)	0.053 (0.071)		0.046 (0.07)	0.049 (0.06)		0.066 (0.103)	0.083 (0.065)		0.085 (0.103)	0.077 (0.063)	
Relative effect size of second strongest variant (%)	40.5 (29.8)	63.6 (51.3)		10.9 (NA)	54.5 (23.5)		34 (20.4)	87.9 (10.4)	0.03	10.9 (NA)	54.5 (23.5)	

MS, maximum statistics method. HT, Hotelling’s *T*^2^ test. LA, LASSO method. CR1% are CR5% the data sets with rare variants using a 1% or a 5% cutoff of allele frequency, respectively. Means and standard deviations are presented. *p*-values are based on a two-group *t*-test. Characteristics that show significant differences in CR1% as well as in CR5% are in boldface.

To further validate our findings, we performed simulations with varying genetic effects of a single causal gene. Nearly independent of sample size, effect size, and number of markers in a gene, the maximum statistics method was better than Hotelling’s *T*^2^ test if there was only one causal variant per causal gene. In contrast, if there were two or more independent variants, the maximum statistics method was outperformed by Hotelling’s *T*^2^ test (results not shown).

## Discussion

In our study, we compared different scoring methods for the combined analysis of common variants and rare variants on the basis of a simulated data set of the GAW17 consortium. The data set was considered representative of genome-wide screening studies that endeavor to select candidate genes for subsequent replication studies. Therefore we focused on gene-wise scoring to define ranked lists of genes for future replication. We considered the ranking of causal genes under variable scoring methods, allele frequency thresholds for collapsing rare variants, and the inclusion or not of rare variants. Scoring methods performed differently in different scenarios. There was no single best method that was superior in all situations. For all phenotypes, either the maximum statistics method or Hotelling’s *T*^2^ test outperformed the other scoring methods.

Our analysis was based on ranking the statistics and selecting a certain number of best-ranked genes instead of calculating and interpreting *p*-values. This approach is equivalent to genomic control [5]. Furthermore, the analysis of ranks rather than *p*-values allows us to make fair comparisons of the scoring methods studied even if the methods differ in the degree of *p*-value inflation. This approach worked well in our analysis in the sense that causal genes were clearly enriched in higher ranks of our gene lists.

However, to be confident of our results, we performed a series of complementary analyses. First, we calculated permutation-test-based *p*-values for each scoring method, analyzing Q1 and Q2 of simulation replicate 1. This procedure yielded interpretable *p*-values and essentially resulted in the same ranking of causal genes as the procedure without permutation (not shown). Second, we repeated most of our analyses applying rigorous quality control. For this purpose, we removed a set of 695 spuriously associated genes from the original GAW17 data set, as proposed by Luedtke et al. [6]. In addition, we adjusted statistics for population stratification and filtered out SNPs that violated Hardy-Weinberg equilibrium. The results were essentially the same as those without quality control (data not shown).

We used the simplest method, proposed by Li and Leal [3], for collapsing rare variants. Numerous more sophisticated collapsing methods are currently available. However, they performed essentially equally well on a gene-wise level for the GAW17 data set (see GAW17, Group 15). A plausible explanation is that only a relatively small number of individuals carried more than one rare variant per gene and that all modeled effects were deleterious.

In our analysis, we collapsed only nonsynonymous rare variants to enrich direct causal effects. In contrast, we used all common variants for analysis, including synonymous common variants. Although only nonsynonymous common variants were causal in the simulation model, synonymous common variants may provide information because of linkage disequilibrium with causal variants. This approach is similar to an experimental design in which a SNP microarray-based study is accompanied by additional sequencing data.

We used four scoring methods to combine the evidence of all markers (common variant and rare variant supermarker): the maximum statistics method, Hotelling’s *T*^2^ test, multivariate analysis, and the LASSO method. Hotelling’s *T*^2^ test and multivariate analysis are established methods specially designed to combine the evidence of correlated tests. In contrast, the maximum statistics method does not consider this correlation structure. The null distribution of the maximum statistics method is a complex order statistic that makes calculating the *p*-values a nontrivial task. We performed no adjustments of the maximum statistics in order to account for the number of markers per gene. This is analogous to selecting single markers in the screening stage of a common multistage genome-wide association study. Furthermore, correcting for the number of markers in a gene did not improve the results of the maximum statistics method in the GAW17 data set (results not shown).

Methodically, the LASSO method can be considered to be in between the maximum statistics method and multivariate analysis because it selects a genetic model first. As shown in Table 1, the LASSO method offered no advantage in our analysis because it was outperformed by either the maximum statistics method or Hotelling’s *T*^2^ test. Among all investigated scoring methods, the LASSO method is the most computationally expensive.

We aimed to characterize conditions under which Hotelling’s *T*^2^ test and the maximum statistics method performed differently. For this purpose, we considered median ranks of causal genes and compared characteristics of those genes for which the ranks differ clearly between methods. We found that the maximum statistics method performed particularly well in genes with a high number of markers but a low number of causal variants (Table 2). This was corroborated by additional simulations that eliminated all possible confounding factors. An explanation for this observation is as follows: Hotelling’s *T*^2^ test statistics are designed to detect several (smaller) deviations from the marginal null hypotheses. This implies that Hotelling’s *T*^2^ test does not perform equally well in detecting single deviations that may be blurred by the noise of other null markers.

## Conclusions

We conclude that the maximum statistics method is a superior scoring alternative to Hotelling’s *T*^2^ test if one expects only one independent causal variant per gene with a dominating effect. Because the true nature of the genetic effect is unknown for real data, both methods need to be taken into consideration. Future work is necessary to investigate how both scoring methods can contribute to the search for genetic modifiers of traits.

## Competing interests

The authors declare that there are no competing interests.

## Authors’ contributions

MS and HK designed the study and performed the statistical analyses. MS wrote the paper. HK contributed to paper writing. All authors read and approved the final version of the manuscript.
